# Levels of Trace Elements in the Lens, Aqueous Humour, and Plasma of Cataractous Patients—A Narrative Review

**DOI:** 10.3390/ijerph191610376

**Published:** 2022-08-20

**Authors:** Zuzanna Micun, Martyna Falkowska, Maryla Młynarczyk, Jan Kochanowicz, Katarzyna Socha, Joanna Konopińska

**Affiliations:** 1 Department of Ophthalmology, Medical University of Białystok, M. Skłodowskiej-Curie 24a, 15-276 Bialystok, Poland; 2 Department of Bromatology, Faculty of Pharmacy with the Division of Laboratory Medicine, Medical University of Białystok, Mickiewicza 2D, 15-222 Bialystok, Poland; 3 Department of Neurology, Medical University of Bialystok, M. Skłodowskiej-Curie 24a, 15-276 Bialystok, Poland

**Keywords:** cataract, microelements, macroelements, cataract nutrition, micronutrients, trace elements in lens, aqueous humour and plasma

## Abstract

Cataracts are one of the most common causes of effective vision loss. Although most cases of cataracts are related to the ageing process, identifying modifiable risk factors can prevent their onset or progression. Many studies have suggested that micro and macroelement levels, not only in blood serum but also in the lens and aqueous humour, may affect the risk of the occurrence and severity of cataracts. This systematic review aims to summarise existing scientific reports concerning the importance of trace elements in cataractogenesis. Many authors have pointed out elevated or decreased levels of particular elements in distinct ocular compartments. However, it is not known if these alterations directly affect the increased risk of cataract occurrence. Further studies are needed to show whether changes in the levels of these elements are correlated with cataract severity and type. Such information would be useful for determining specific recommendations for micronutrient supplementation in preventing cataractogenesis.

## 1. Introduction

A cataract is an opacification of the lens that obscures the passage of light to the retina of the eye, leading to a decrease in vison. It is the main cause of reversible blindness with an estimated 95 million people affected worldwide [1,2]. Studies indicate that over 12 million people are blind worldwide due to cataracts [3]. Although cataracts most commonly develop due to ageing and UV exposure, several risk factors have been identified, including many systemic disorders, e.g., diabetes mellitus, hypertension, chronic kidney disease, autoimmune disease [4], and systemic use of corticosteroids [5]. Identifying modifiable risk factors for this eye condition, such as nicotine consumption, alcohol intake, and ultraviolet light exposure, can prevent the onset or progression of cataracts [4,6]. Moreover, it should be noted that some environmental factors such as IOP-lowering medications/surgery, trauma, steroid usage, and certain occupations may increase the rate of this disease. The diagnosis is based on a slit-lamp examination after pupillary dilation and confirmation of the objective criteria for cortical, nuclear, and posterior subcapsular cataracts are described, followed by typical symptoms such as decreased visual acuity, contrast sensitivity and foggy vision, altered colour perception, decreased mesopic and scotopic vision, glare, myopisation, and monocular diplopia [2]. Diagnostic evaluation consists of a general ophthalmic examination, including history, refraction and vision testing, tonometry, and morphologic examination of anterior and posterior segment, and is supplemented by special tests and examinations, such as interference vision (retinometer), keratometry including corneal topo-/tomography, biometry (ultrasound and optical), specular microscopy of the corneal endothelium, and OCT of the central retina [2].

Treatment options at earlier stages, such as correction with refractive spectacles, pupillary dilatation, and a few pharmaceutical eye drops, allow patients to carry on everyday activities. Once the cataract has become advanced, treatment is exclusively surgical: evaluating the indication for surgery individually; weighing chances and risks; determining individual decisions such as anesthesia, surgical technical options, target refraction, and intraocular lens options [2]. The procedure of choice is phacoemulsification. In this method, the opaque lens is removed and replaced by an artificial intraocular lens, with very high effectiveness in restoring sight and early visual recovery [1].

There are scientific reports on the influence of levels of selected trace elements on the risk of different eye diseases’ onset and progression [7,8]. Only a few review papers [4,9,10] have focused on the level of trace elements, e.g., iron, calcium, zinc, and selenium, and their connection with the onset and progression of cataract development. There is a lack of research discussing the contribution of all components and thus providing specific guidelines for the patient. This article reviews the available publications on micro and macroelements levels, not only in the blood serum but also in the lens and aqueous humour, and their contribution to cataractogenesis, with special attention paid to specific dietary guidelines for the patient.

## 2. Sodium and Potassium

### 2.1. The Role in Cataractogenesis

The levels of sodium (Na) and potassium (K) in the human lens are established by the action of sodium–potassium adenosine triphosphatase (Na^+^/K^+^-ATPase). Its role is fundamental in balancing ion transport in both directions. Na^+^/K^+^-ATPase activity is different in the epithelial cells and fibres of the lens, though it is assumed that the epithelium-mediated ion transport contributes significantly to the regulation of ionic composition in the entire lens [11]. A cation imbalance might result in osmotic disturbances and water accumulation in lens cells and eventually lead to cell lysis and the appearance of fluid droplets that scatter light and impair transparency [12]. The activity of Na^+^/K^+^-ATPase may decrease due to ageing, oxidation, and glycation. Na^+^/K^+^-ATPase functioning might be insufficient in the cataractous lens due to changes in the enzyme kinetics [13]. Most studies suggest that Na^+^/K^+^-ATPase activity is reduced in cataracts [13,14,15,16,17]. However, other studies have shown that Na^+^/K^+^-ATPase activity in human cataracts was unchanged [18,19,20] or even increased in some cases [21]. It has been proposed that membrane permeability may be abnormally high in cataractous lenses [19,22]. Na^+^/K^+^-ATPase overloaded by increased plasma sodium levels might be unable to maintain the low levels of intracellular sodium required for lens transparency. That could explain why patients with cataracts have higher lens sodium levels than those without [23].

### 2.2. Studies In Vivo

Many researchers have demonstrated notably elevated lens sodium in age-related cortical cataracts and diabetic cataracts [24,25,26,27,28]. A number of studies have found that alterations in the cation concentration of the aqueous humour remain correlated with the serum cation concentration, so it seems that diets with a high Na^+^ content might be a risk factor for age-related cataract formation. The Blue Mountains Eye Study [23] found a relationship between a high dietary sodium intake and posterior subcapsular cataracts and suggested that a reduced salt diet may help prevent cataract development among the elderly. Similar conclusions were also drawn by other authors [12,29,30,31,32,33,34]. Na^+^/K^+^-ATPase is also responsible for maintaining the correct concentrations of potassium in the lens. Donnelly [35] found an association between cuneiform cataracts and a reduced plasma potassium level. On the other hand, raised potassium levels appear connected with mature/hypermature cataracts. Dilsiz et al. [27] confirmed that the lens potassium level is decreased in several types of cataracts compared with normal lenses. This study supports the hypothesis that damage to lens cell membranes affects the ion exchange mechanism in age-related cataracts. Comparable conclusions were made in other studies [26,28]. According to Mansour et al. [29], variations in the mean serum K+ level in senile cataract patients and normal individuals were statistically insignificant.

## 3. Magnesium

### 3.1. The Role in Cataractogenesis

Magnesium (Mg), an important regulatory cation involved in many biological processes, is known to be relevant for maintaining the structural and functional integrity of the lens. It plays a significant role as a cofactor for enzymes utilising ATP in the human body. It is crucial in regulating the intracellular ionic environment and for proper functioning of magnesium-dependent enzymes involved in adenosine triphosphate (ATP) production and hydrolysis [36]. Imbalances in the intracellular ionic concentrations of cataractous lens might develop due to membrane permeability changes, as described by various researchers.

### 3.2. Studies In Vivo

Dilsiz et al. [27] found a strong association between the development of age-related cataracts and decreased magnesium and potassium of the lens coupled with their increased calcium and sodium content. Magnesium deficiency was noted to be the cause of ionic imbalances; in particular, it leads to ATP depletion and dysfunction of membrane-associated transporters, resulting in reduced intracellular K^+^ and increased Na^+^ and Ca^2+^ [37,38]. Ca^2+^ ATPase is a magnesium-dependent membrane-associated transporter that regulates the intracellular ionic lens environment by removing any excess of calcium [39]. Magnesium deficiency alters membrane-associated ATPase functions and leads to calcium accumulation in the lens, eventually resulting in opacification [37]. Magnesium deficiency triggers increased inducible nitric oxide synthase (iNOS) expression and the release of nitric oxide, which has been shown to play a critical role as it produces nitrogen free radicals that are capable of causing oxidative damage [36]. Kao et al. [40] showed that nitric oxide (NO) levels in the aqueous humour are higher in patients with cataracts, and the extent of this rise is correlated with age and cataract maturity. Increased nitrite contents have been shown in human lenses with posterior subcapsular cataracts [41]. Moreover, nitrite ions have been shown to react directly with crystalline, resulting in its modification in a manner similar to that occurring in ageing lenses and cataracts. This non-enzymatic nitration of crystalline may also be an important mechanism contributing to cataractogenesis [42]. Increased systemic oxidative stress generated outside the lens can be an important factor leading to cataract formation, and magnesium deficiency might contribute to its escalation. Altered plasma antioxidant parameters are shown to reflect increased oxidative stress in cataract patients. The oxidative stress resulting from Mg deficiency might be destructive and lead to lens fibre apoptosis, further contributing to cataractogenesis [37,43]. These data allow us to connect the alterations in lenticular redox status and ionic imbalances with magnesium deficiency in cataract pathogenesis. Studies in rats performed by Shumiya [44,45] demonstrated the preventive role of magnesium in cataract development. It was observed that a low magnesium diet accelerates cataract development, whereas magnesium supplementation might delay the onset of cataracts in vivo. Nevertheless, the association of magnesium deficiency with human age-related cataracts can only be considered an associated factor, along with several other factors. The scientific evidence is lacking regarding the role of magnesium supplementation in delaying the onset or slowing the progression of cataracts in humans.

## 4. Calcium

### 4.1. The Role in Cataractogenesis

Calcium (Ca) has long been known to play a role in cataractogenesis. The first to appreciate its contribution to cataract development was Burge [46], who demonstrated that cataractous human lenses tended to have a much higher calcium content than normal lenses. More recent studies have confirmed this correlation [24,26,27,28,47,48,49,50,51,52]. There is reasonable evidence that aqueous humour calcium levels precisely reflect the level of free calcium in the plasma. It is important to determine whether the variability in the calcium content of the aqueous humour is specific to natural ageing, or if it is strictly associated with cataracts, and how it affects the calcium concentration in the lens [53]. The inward passive diffusion of calcium is offset by the actions of calcium adenosine triphosphatase (Ca^2+^-ATPase). The increased entry of calcium into clear lenses that occurs with advance age is countered by an amplification in the activity of Ca^2+^-ATPase [54]. With cataracts, Ca^2+^-ATPase activity is decreased by 50% and lens membrane permeability further increases [19,22,55,56,57], resulting in elevated total lens calcium levels. Moreover, Ca^2+^-ATPase is sensitive to lipid order, which changes with age and in cataracts [58].

### 4.2. Studies In Vivo

As early as 1929, Dorothy Adams [59] noticed that many patients presenting with cataracts had a higher plasma calcium concentration than their ophthalmologically normal counterparts. She assumed that the aqueous humour calcium levels were likely also higher. Her study involved incubating bovine lenses in solutions with a higher calcium content than the normal aqueous humour, resulting in the formation of light-scattering opacities. Experiments carried out by Becker et al. [60] showed that removing calcium from the lens caused widespread disruption of the internal ion levels, and this in turn led to lens swelling and opacification. An intriguing problem is therefore why calcium, both in excess and in deficit, can induce cataracts. There are several reasons an increase in internal calcium may cause lens opacifications. A high lens calcium level changes the organisation of proteins within the membrane gap junctions, which in turn impedes the intercellular exchange of substances and may result in cataract formation [61]. In lenses with higher than moderate calcium concentrations, protein aggregation can occur [62]. Dense precipitates of calcium salts (calcium phosphate and calcium oxalate) tend to coexist with extremely high calcium concentrations [63]. A correlation between calcium and sodium levels in the lens is observed. Duncan et al. [57] found that lenses with a high calcium concentration also had a high sodium content. Highly localised opacities were found in lenses with the highest calcium concentrations and near normal sodium levels, while a low calcium content was associated with nuclear cataracts. The same study postulated that calcium is implicated in decreases in protein synthesis and an induction of net protein loss. This could lead to disturbances in the nature of the homogeneous cell cytoplasm, which might result in a marked increase in light scattering. Since it has been increasingly recognised that cataracts are associated with medical conditions that involve hypocalcaemia [64], the emphasis has shifted towards investigating the role of calcium in maintaining transparency. Among other well-known risk factors, osteoporosis is associated with the presence of cataracts. Nemet et al. [65] connected cataracts with osteoporosis and considered calcium an important factor in the development of both diseases. In all age groups of their study, osteoporosis was more prevalent in cataract patients than in the control group [66].

## 5. Iron

### 5.1. The Role in Cataractogenesis

The involvement of iron in cataract formation has been considered in many studies [67,68,69,70,71,72,73,74,75,76,77,78,79,80,81]. Its role in cellular metabolism, based on the iron-catalysed formation of reactive oxygen species (ROS), leads to oxidative damage of the lens [67]. Many redox reactions in cells deliver oxygen radical superoxide, detoxified by superoxide dismutase, resulting in the formation of hydrogen peroxide, which in the presence of ferrous iron can form highly reactive and damaging hydroxyl radicals. These mechanisms play a significant role in the pathophysiology of numerous diseases, including cataracts [67,68,69,70]. Iron-catalysed reactions have been proven to be a key factor in lens DNA damage [71] and changes in lens crystallines [72,73]. It thus becomes relevant to determine if iron levels and reactivity change in the lens during cataractogenesis.

### 5.2. Studies In Vivo

Research by McGahan [74] found that the lens has noticeable control over its iron content. Goralska et al. [75] suggested that the lens not only has the ability to reduce potentially toxic intraocular iron levels but also precisely controlled mechanisms for maintaining iron levels within a narrow range. After the breakdown of the blood–ocular barriers during inflammation, the lens is able to collect iron from increased plasma transferrin and non-transferrin-bound iron present in the intraocular fluids. Moreover, the iron concentration of the lens declines to primary levels after termination of the inflammatory process. It is very likely that these mechanisms change with age, leading to increased levels of iron in the lens. This may result in the oxidative damage observed in cataractogenesis. Sixto García-Castiñeiras [76] found that transferrin was specifically enriched in the aqueous humour of senile cataract patients in proportion to cataract severity. Their study was based on the internalisation and lysosomal destruction of ferroportin located in the basolateral membranes of the intestinal absorptive cells in a process called anaemia of inflammation. The disappearance of ferroportin could result in iron accumulation within the cytoplasm of cells exporting iron through this transporter, leading to increased intracellular oxidative stress in these cells [77]. However, further research of this mechanism is needed. Dawczynski et al. [78] found iron uptake rates were higher in adult lenses, particularly those with advanced-stage cataracts. This may be related to ferritin degradation or ultraviolet A exposure [79]. The same study showed higher levels of redox-active iron (not bound with ferritin) in cataractous compared to noncataractous lenses. In the lens opacities case/control study of Leske et al. [80], iron intake, as judged by the amount and type of food ingested, was evaluated, resulting in the claim that iron represents a protective factor against cataracts. Compared with nondiabetic subjects, Aydin et al. [81] did not find significantly increased levels of iron in the blood, aqueous humour, or cataractous lenses of diabetic patients.

## 6. Selenium

### 6.1. The Role in Cataractogenesis

Age-related cataracts are mostly associated with oxidative stress, due to the presence of reactive oxygen species (ROS) [82]. The leading factor protecting the lens against ROS-induced damage is glutathione/glutathione peroxidase 1 and 4 (GSH/GPX1/GPX-4), known as selenoproteins because of their selenium-dependent activity [83]. Selenium (Se) present in the form of selenocysteine in a GPX molecule additionally increases its enzymatic activity almost 1000-fold compared with cysteine homologues [84]. GPX reduces the increased level of hydrogen peroxide H_2_O_2_ present in the aqueous humour of cataract patients [85].

### 6.2. In Vitro and In Vivo Studies

Selenium is an essential micronutrient for humans. Much of selenium’s beneficial influence on health is attributed to its presence within 25 selenoproteins [83]. Previous studies showed that the supplementation of selenium could slow the development of naphthalene cataract, possibly by attenuating the oxidative stress in the lens [84]. However, the mechanism of selenium in preventing or slowing cataract onset and progression remains virtually unclear [83].

There are reports suggesting that insufficient Se levels may negatively affect lens metabolism, increasing the opacity [81,86,87,88]. Research by Post [89] confirmed the association between low serum selenium levels and age-related cataracts and suggested that it may constitute a potential risk factor for both nuclear and cortical age-related cataracts. Dawczynski’s study [90] showed that increasing lens opacification and coloration is connected with a significant increase in the selenium content of lenses. In his research, patients with a mature cataract showed higher selenium levels in the lens compared to those with other cataract forms, with the opposite trend for blood serum. Flohé [86] claimed that selenium is an integral part of glutathione peroxidase type 1; it prevents oxidative damage and, consequently, cataract formation in the eye lens, although there is no evidence that any selenium supplementation that exceeds the dietary reference intakes can be recommended for age-related cataract prevention. Moreover, according to his research, any amount of selenium that exceeds the tiny amounts required for selenoprotein synthesis is toxic and can even cause cataracts, as has been shown in experimental animals.

## 7. Zinc

### 7.1. The Role in Cataractogenesis

Many researchers have attempted to establish the association between age-related cataracts and zinc (Zn) levels in the lens. Zinc ions are directly absorbed from the aqueous environment and accumulate mostly in the metabolically active cortex region rather than the nucleus section of the lens [91]. Zinc is directly involved in vision, and its deficiency may hinder dark adaptation [92]. On the contrary, increased zinc levels might result in the oxidation of sulfhydryl groups or increased metallothionein levels, eventually leading to reduced permeability of the lens membrane. Excess zinc in the lens might be linked with the presence of high molecular weight proteins, which are considered to be precursors of insoluble protein aggregates that cause lens opacification [89]. It is not understood whether the high levels of zinc in cataractous lenses result from the presence of metalloproteins or from the association of metals with small molecules [93].

### 7.2. Studies In Vivo

Dawczynski et al. [78] demonstrated the correlation between lens coloration intensity and elevated lens zinc content, specifically associated with advanced forms of cataracts and dark brown coloured lenses. Moreover, an increased zinc level in cataractous lenses was found by Rasi et al. [48]. Stanojević-Paović et al. [26] found that zinc concentrations in cataractous lenses were much higher than those in the aqueous humour. In addition, the Zn levels in the aqueous humour were much lower compared to those in healthy subjects. The study by Soares et al. [94] showed that even though zinc deficiencies in plasma or erythrocytes are common among the elderly, there does not seem to be any significant dependence between the zinc level and the risk of cataract occurrence. Gündüz et al. found zinc concentrations were increased in the lens of diabetic patients compared with the nondiabetic group, suggesting that zinc might play a role in cataract pathogenesis of diabetic senile individuals [95]. The Linxian studies indicate that the prevalence of nuclear cataracts is reduced by supplementation with retinol/zinc [96].

## 8. Copper

### 8.1. The Role in Cataractogenesis

Several mechanisms are crucial for maintaining lens transparency, including those associated with peroxidation. It is known that a decrease in the reactivity of the copper-containing enzyme superoxide dismutase and an increase in hydrogen peroxide concentrations lead to the generation of hydroxyl radicals from Fenton-type reactions. Hyperactivity of the peroxidation cascade, based on the release of copper ions from copper-containing enzymes due to hyperglycaemia in diabetics’ lenses, may result in elevated copper (Cu) levels. This mechanism may lead to lenticular opacification due to damaging of proteins, lipids, and membranous structures [97]. Nevertheless, the current literature lacks information on the above presented mechanisms in nondiabetic patients, and the correlation between copper and cataracts is still yet to be evaluated.

### 8.2. Studies In Vivo

Lin [97] reported that the concentration of copper ion is higher in cataractous lenses than in clear lenses. Moreover, copper ion levels are significantly higher in subjects with diabetes than those without. Similar conclusions were drawn by Aydin et al. [81]. Some authors have detected raised levels of Cu in cataractous lenses [28,98,99,100,101]; however, Swanson et al. found decreased levels of Cu in cataractous lenses [102], whereas Cooks et al. found no correlation between Cu and cataracts [103].

## 9. Toxic Elements

Several studies have attempted to demonstrate the correlation between age-related cataracts and elevated levels of potentially toxic trace elements. Dolar-Szczasny et al. aimed to measure variations of trace element levels in the aqueous humour and showed that patients with cataracts are characterised by elevated levels of very toxic elements in the anterior chamber fluid, e.g., thallium, tellurium, and caesium. The results obtained for increased levels of phosphorus, lead, and aluminium in cataract lenses were also clinically relevant in consideration of their neurodegenerative potential [104]. Furthermore, low-level lead exposure may be an important risk factor for the development of age-related cataracts. Lead is successively mobilised from the skeleton and circulates in plasma at very low levels, where it is available for interactions with other tissues. It can interfere with lens epithelial cells and may disrupt lens redox status and cause protein conformational changes that decrease lens transparency [105]. The presence of lead in lenses with cataracts has been determined in several studies. Moreover, the lead level was shown to be elevated in cataractous lenses compared with clear lenses [28,98,99,106,107]. In addition, Shukla N et al. [28] indicated that lens lead levels were inversely correlated with lens levels of the antioxidant zinc. Lens transparency may be affected by the intrusion of lead into the lens, which causes changes in protein conformation. Debra A Schaumberg suggested that the reduction of lead exposure could help decrease the global burden of cataracts [105].

## 10. Cataracts and Special Diets

The ketogenic diet (KD) is a high-fat, low-carbohydrate, and adequate-protein diet regime, which has gained a popularity as an adjuvant therapy in many clinical conditions especially in the context of neurological diseases (NDs) [108,109]. There are many types of ketogenic diets, among which the classic long-chain triglyceride (LCT) KD which uses long chain triglyceride as its primary fat source, and the medium chain triglyceride (MCT) diet which contains more carbohydrate and protein as a result of increased ketogenic potential of MCT are the most commonly used [110,111]. Although KDs could be beneficial for many patients suffering from neurological, metabolic, or even oncological disorders, KD as a restrictive dietary treatment promotes a risk of nutritional deficiency [112,113]. As a Christodoulides’s 12 month randomised trial suggests, the plasma levels of zinc, selenium, and magnesium in the KD children are more likely to be low [114]. The results of a Prudenico study show that KD causes multiple deficiencies of vitamins and minerals, e.g., calcium, magnesium, and phosphorus, which indicate inadequate dietary intake in all patients. A similar conclusion, with regard to calcium, phosphorus, magnesium, iron, copper, and zinc was found by Zupec-Kania and Zupanc [115], and to selenium by Bergqvist [116].

The vegetarian diet is attributed to a food consumption pattern which excludes meat and fish and uses, instead, plant-origin products. There are various forms of vegetarianism, among which veganism is the strictest one [117]. Many researchers study those diets to determine the health benefits and disadvantages of following them, including assessing the amount of micro and macronutrients provided by their use [118,119]. The Neufingler and Eilander review [118], opposite to the Alles’s one [120], shows that the average calcium intake in vegetarians is higher than in vegans or meat eaters [118]. In addition, iron and magnesium intakes tend to be higher in plant-based eaters than meat eaters [118,119,120,121]. Bakaloundi’s results suggest that vegans also consume lower amounts of sodium, potassium, selenium, and zinc with their diet and a higher amount of copper than others [119].

There is no general definition of a high-protein diet. However, in the food industry the term ‘protein enriched’ products can be used where at least 20% of the energy value of the food is provided by protein [122]. In Gwin analyses from 2019, a higher protein density diet is associated with greater diet quality and higher intake of micronutrients such as potassium, calcium, magnesium, iron, and zinc [123]. Moreover, the Hunt study showed that a diet enriched in protein provided by meat improved calcium absorption from the low-calcium diet and did not change its absorption in the case of a high-calcium diet [124].

The majority of dietary intake studies suggest that reduction in the risk and progression of cataracts is obtained with diets high in vitamin C, E and A, carotenoids, and selenium [125]. However, Christin randomized trial data indicate that daily selenium supplementation in a large cohort of men was unlikely to have a significant effect on age-related cataracts [126]. On the other hand, observational data from the AREDS show that using a rich in magnesium, iron, copper, zinc, and vitamins Centrum multivitamin may delay the progression of lens opacities [127]. Linxian cataract studies present that a daily intake of vitamin/mineral supplements may also decrease the risk of nuclear cataracts [97].

## 11. Discussion

The structural and functional integrity of the lens, which is of primary importance in transmitting light to the retina, largely depends on the maintenance of intracellular and extracellular ionic homeostasis. The strong association between cataract formation and cigarette smoking may suggest that the accumulation of metal ions is relevant to pathogenesis. Limited intake and higher elimination of nutrients related to the prevalence of chronic diseases, drug therapy, and unbalanced dietary habits among the elderly increase the risk of developing trace element deficiencies. This may lead to the disruption of homeostasis and result in the development of numerous diseases, such as age-related cataracts.

The evidence provided throughout this review shows that there is a substantiated correlation between specific element levels, i.e., sodium, potassium, magnesium, calcium, iron, selenium, zinc, copper, lead, etc., in the lens, aqueous humour, and serum associated the pathogenesis of age-related cataracts. These micro and macroelements participate in a number of metabolic pathways in the human body. They regulate each other’s content in different compartments and, hence, their excess or deficiency have various multidirectional implications. The authors quoted here have pointed out that disturbances in elemental contents play a role in cataractogenesis by having an indirect effect on oxidative stress, ion pump activity, inflammatory processes, and transcellular transport. The available research on the relationship between macro and microelements and cataracts focuses primarily on element levels within the lens. More studies are needed to evaluate these correlations in the aqueous humour and serum as well.

Although cataract surgery is a safe and highly effective treatment resulting in a best-corrected visual acuity of 20/40 in 95% patients after surgery [9], factors predisposing patients to the occurrence and progression of cataracts are still relatively difficult to determine. Identifying these factors could potentially reduce cataract incidence and related complications in addition to cutting down on costs associated with poor vision, such as treatment of depression, injuries, hospital admissions, and the need for caretakers [10].

Despite its value, our review has some limitations. Research included in the analysis differed both in terms of inclusion criteria and the number of test subjects. Additionally, in some articles, the age of the tested patients was not precisely specified, which could have had a significant impact on the results considering the fact that many of the abovementioned pathomechanisms evolve with age. Moreover, many studies were conducted on animals, and we are uncertain whether the patterns and metabolic processes in animals are exactly the same as in humans. The topic could be explored in more detail in a meta-analysis that takes into consideration the age of the patients and concomitant medical conditions that may lead to disturbances in the body’s micronutrient and macronutrient levels, as well as an assessment of the diet and eating habits of those included in the study.

## 12. Conclusions

The abovementioned reports regarding different micro and macroelements and their role in cataract pathogenesis demonstrate the complexity of its aetiology and allow us to better understand it. Extending clinical studies on the levels of these components in the plasma, aqueous humour, and lens, and consideration of indications for their supplementation or restriction, may prove to be a useful tool in the prevention of cataracts. Up to date, there is no clear dietary recommendation regarding the topic of cataract onset and progression and certain trace elements. Knowledge in this area may also serve as a valuable adjunct to disease treatment. However, randomised trials conducted on large cohorts are still needed to improve our knowledge and standardise the specific indications for appropriate nutrient supplementation.

## 13. Methods of Literature Search

**Figure 1:**
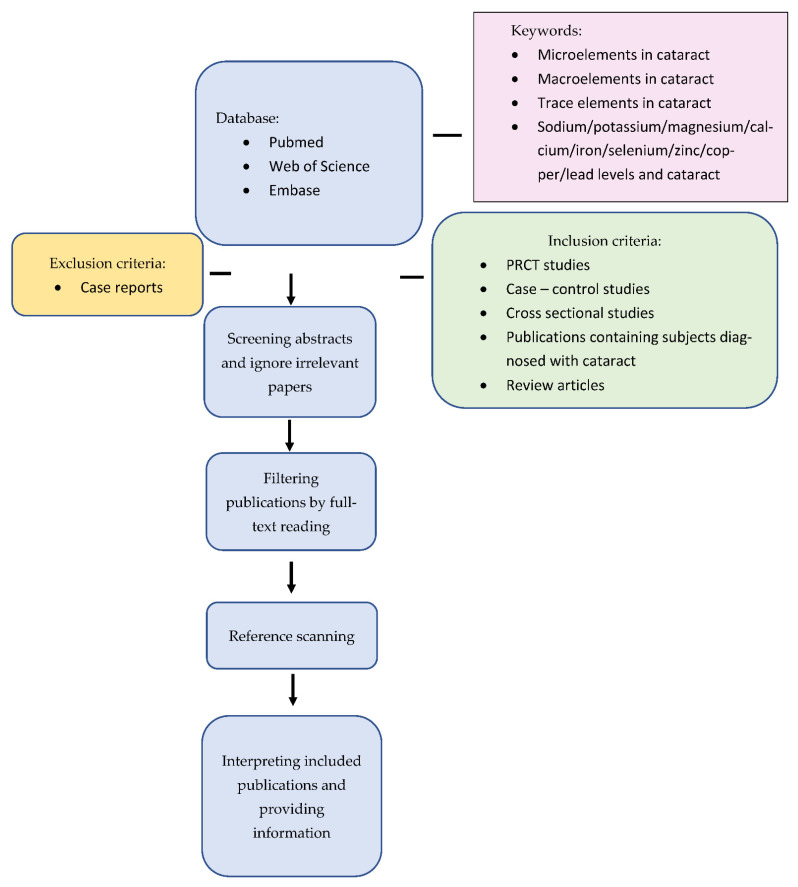
Flowchart showing the proposed approach.

For the purpose of this narrative review, on the 15th of June a search was undertaken of the PubMed, Web of Science, Scopus, and Embase databases, concerning the prevalence of age-related cataracts and levels of trace elements in lens, aqueous humour, and plasma. Search strategy terms included “microelements AND cataract”, ”macroelements AND cataract”, “trace elements AND cataract”, and the phrase “cataract” combined with all microelements mentioned in this review (sodium, potassium, copper, magnesium, calcium, iron, selenium, and zinc). The subsequent search with the phrase “lens opacities” combined with the abovementioned terms was repeated. Published articles in English were preferentially selected based on the compatibility of abstracts with the topic of the study. Additionally, Google Scholar was used to access gray literature. In addition, a cataract expert was consulted to identify important articles. Articles inconsistent with the topic and abstracts available only as conference papers were excluded. Relevant in-article references not returned in our searches were also considered. We included prospective randomised control trials, case–control studies, cross-sectional studies, review articles, and publications with patients with a diagnosis of cataracts with no year range for all articles (Figure 1).

All the extracted papers from each database were inserted in Endnote X6, and duplicates were removed. Then, the screening was completed independently by the two authors (Z.M. and J.K.). In the first step, the titles were reviewed, and if the article was relevant, then the abstract and then the full text of the article was reviewed. In the case of any discrepancies, the third person’s opinion (M.M.) was sought (Table 1, Table 2 and Table 3).

In this way, 425 full-text original articles, reviews, randomised clinical trials, retrospective and cohort studies were included in the study. We included in vitro research, research on laboratory animals, and human clinical trials. Because the journal guidelines restrict the number of references, we chose those studies which seemed relevant in our opinion.

### Exclusion Criteria

In this study, we only reviewed levels of trace elements in age-related cataracts, not connected with specific ocular or systemic diseases (e.g., Down syndrome, glaucoma, arthritis, and diabetes). Exclusions were also applied to other types of cataracts, including acquired cataracts due to trauma or medications, congenital cataracts, and other types of secondary cataract due to certain ophthalmic diseases such as uveitis. Publications such as letters, conference papers and abstracts, notes, editorials, clinical studies, follow-up, and longitudinal studies were also excluded. All studies that did not mention their methods, and studies that used self-report or a questionnaire for the diagnosis of cataracts, were excluded.

## Figures and Tables

**Table 1:** Intake/exposure of selected trace elements and associated risk of developing cataracts.

References	Examined Factor	Likely Impact on Risk of Developing Cataracts
[12,23,29,30,31,32,33,34]	High sodium intake	↑
[33,45]	Low magnesium intake	↓
[64,65]	Low calcium intake	↑
[80]	Low iron intake	↑
[86]	High selenium intake	no evidence
[96]	Low zinc intake	↑
[105]	High lead exposure	↑

**Table 2:** Levels of selected micro and macroelement and associated risk of developing cataracts.

Element	References	Examined Factor	Risk of Developing Cataracts
Sodium	[24,25,26,27,28]	High lens level	↑
	[12,23,29,30,31,32,33,34]	High plasma level	↑
	[29]	High aqueous humour level	↑
Potassium	[26,27,28]	Low lens level	↑
	[35]	Low/High plasma level	↑
Magnesium	[27,36,37,38]	Low lens level	↑
Calcium	[24,26,27,28,46,47,48,49,50,51,52,57,61,62,63]	High lens level	↑
	[60]	Low lens level	↑
	[59]	High plasma and aqueous humour level	↑
Iron	[76,77,78]	High lens level	↑
Selenium	[81,86,87,88]	Low lens level	↑
	[90]	High lens level	↑
	[89]	Low plasma level	↑
Zinc	[48,89]	High lens level	↑
	[26]	Low aqueous humour level	↑
	[94]	Low plasma level	no impact
Copper	[28,81,97,98,99,100,101]	High lens level	↑
Toxic elements	[104]	High aqueous humour thallium, tellurium, caesium, lead, aluminium, phosphorus level	↑
	[28,98,99,105,106,107]	High lens lead level	↑

**Table 3:** Levels of selected micro and macroelement and associated risk of developing cataracts depending on compartment.

Compartment	Examined Factor	References	Risk of Developing Cataracts
Lens	High sodium level	[24,25,26,27,28]	↑
	Low potassium level	[26,27,28]	↑
	Low magnesium level	[27,36,37,38]	↑
	High calcium level	[24,26,27,28,46,47,48,49,50,51,52,57,61,62,63]	↑
	Low calcium level	[60]	↑
	High iron level	[76,77,78]	↑
	Low selenium level	[81,86,87,88]	↑
	High selenium level	[90]	↑
	High zinc level	[48,89]	↑
	High copper level	[28,81,97,98,99,100,101]	↑
	High lead level	[28,98,99,105,106,107]	↑
Plasma	High sodium level	[12,23,29,30,31,32,33,34]	↑
	Low/High potassium level	[35]	↑
	High calcium level	[59]	↑
	Low selenium level	[89]	↑
	Low zinc level	[94]	no impact
Aqueous humour	High sodium level	[29]	↑
	High calcium level	[59]	↑
	Low zinc level	[26]	↑
	High thallium, tellurium, caesium, lead, aluminium, phosphorus level	[104]	↑

## Data Availability

All materials and information will be available upon an e-mail request to the corresponding author.
